# Chemically driven negative linear compressibility in sodium amidoborane, Na(NH_2_BH_3_)

**DOI:** 10.1038/srep28745

**Published:** 2016-06-30

**Authors:** Ewelina Magos-Palasyuk, Karol J. Fijalkowski, Taras Palasyuk

**Affiliations:** 1 Institute of Physical Chemistry, Polish Academy of Sciences, Kasprzaka Str. 44/52, 01-224 Warsaw, Poland; 2 Center of New Technologies, University of Warsaw, S. Banacha Str. 2c, 02-097 Warsaw, Poland

## Abstract

Over the past few years we have been witnessing a surge of scientific interest to materials exhibiting a rare mechanical effect such as negative linear compressibility (NLC). Here we report on strong NLC found in an ionic molecular crystal of sodium amidoborane (NaAB) – easily-accessible, optically transparent material. *In situ* Raman measurements revealed abnormal elongation of B-N and N-H bonds of NaAB at pressure about 3 GPa. *Ab initio* calculations indicate the observed spectroscopic changes are due to an isostructural phase transition accompanied by a stepwise expansion of the crystal along *c* axis. Analysis of calculated charge density distribution and geometry of molecular species (NH_2_BH_3_) univocally points to a chemically driven mechanism of NLC – pressure-induced formation of hydrogen bonds. The new H-bond acts as a “pivot screw” coupling N-H covalent bonds of neighbor molecular species – a system resembling a two-lever “jack device” on a molecular scale. A mechanism based on formation of new bonds stands in apparent contrast to mechanisms so far reported in majority of NLC materials where no significant alteration of chemical bonding was observed. The finding therefore suggests a qualitatively new direction in exploration the field towards rational design of incompressible materials.

Negative linear compressibility (NLC) defines an uncommon property of material to expand along at least one direction when subjected to uniform external pressure. Despite the counterintuitive character the phenomenon does not violate basic thermodynamic principle of overall volume reduction under pressure and may lay foundation for development of effectively incompressible materials for various practical applications, including materials for artificial muscles, amplification of piezoelectric response in sensors and actuators, tunable sieves for filtration, “smart” body armor made of robust shock absorbing materials, sensitive pressure detectors, materials used at deep sea, etc. The most significant challenges in this field have been the apparent rarity of materials showing NLC, the extreme weakness of the NLC effect found in these materials and little understanding of mechanisms governing NLC in particular materials^1^,^2^,^3^. Examples of NLC were reported in extensive inorganic (or organic), metal-organic as well as molecular framework materials^4^,^5^,^6^,^7^. In these materials the NLC responses tend to occur without breaking the symmetry of crystal. Examples of reversible NLC in simple inorganic compounds remain extremely rare and those reported exhibit NLC mostly as a result of a stepwise symmetry breaking phase transition^8^,^9^,^10^,^11^. To the best of our knowledge there is no report on a simple system where NLC induces no symmetry change to crystal. So far in search of anomalous mechanical response to external pressure the main strategy was to exploit materials with specific geometric motifs, e.g. “wine-rack”, inverse honeycomb, helices, etc., that inherently favour NLC. In the majority of known materials NLC behaviour is conditioned roughly by a geometric effect involving no significant change to bonding interactions in material. The latter observation can be also applied to methanol monohydrate, which is, to the best of our knowledge, the only NLC material involving hydrogen bonds^12^. Here we report on an example of a relatively simple inorganic molecular compound, sodium amidoborane, exhibiting unexpectedly strong NLC with no symmetry change across the phase transition. Moreover, here we show sodium amidoborane is an example of material with chemically mediated NLC – via reversible formation of new hydrogen bonds – a mechanism which has been never reported in other incompressible materials.

Sodium amidoborane (NaNH_2_BH_3_, NaAB) has recently attracted considerable interest as a potential candidate material for hydrogen storage^13^. In general amidoboranes (MeAB) are ammonia borane (NH_3_BH_3_, AB) derivatives obtained by substitution of a hydrogen proton in the NH_3_ group for a metal cation. Amidoboranes of alkali metals (Li, Na, K) release ca. 10 wt.%, 6 wt.% and 4 wt.% of molecular hydrogen respectively, and in contrast to pure AB, hydrogen is released in a quasi one-step process, at temperatures below 100 °C. This significantly improved dehydrogenation profile indicates substantial difference in decomposition mechanisms of AB and its metallated derivatives, most probably, as a result of different bonding. Applying hydrostatic pressure may induce substantial alteration of interatomic distances therefore providing convenient tool for probing intra- and intermolecular interactions. As an example, in our recent work on potassium amidoborane we found that weak intermolecular interactions experienced significant enhancement under pressure^14^. It is in this context we have choosen to study sodium amidoborane using diamond anvil cell technique coupled with Raman spectroscopy for *in situ* probing vibrational properties under pressure.

## Results and Discussion

**Figure 1:**
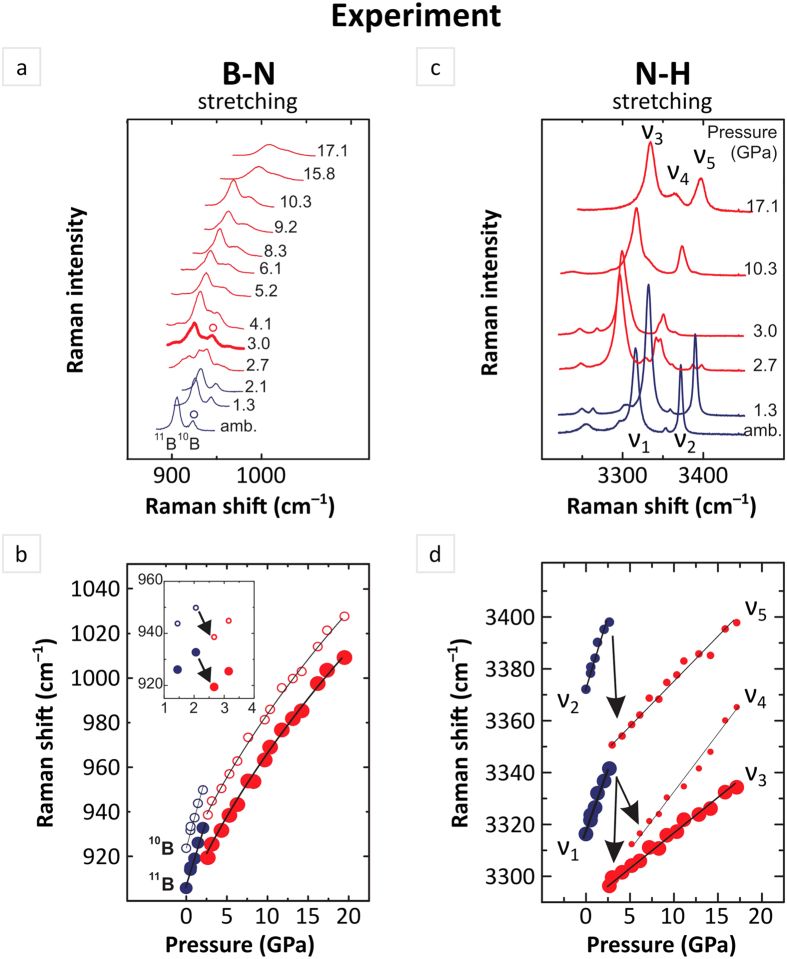
Experimental Raman spectra of Na(NH_2_BH_3_) and analysis of pressure effect on frequency of corresponding modes. (**a**) Frequency range of B-N stretching modes. Spectra collected at different pressures are offset for clarity. (**b**) Change of frequency of B-N stretching vibrations with pressure. Size of symbols corresponds to intensity of measured signal. Lines are for eye-guide only. (**c**) Frequency range of N-H stretching modes at different pressures. Weak signals present in spectra are related to trace amount of unreacted ammonia borane (NH_3_BH_3_). (**d**) Pressure dependence of Raman frequencies of N-H stretching vibrations, assigned as v_1–5_ for clarity.

Freshly synthesized samples of NaAB were used for our spectroscopic study. Details of synthesis procedure and variable pressure Raman measurements are described in Methods. Here we will focus on changes observed in the spectral range of B-N (skeletal) as well as N-H stretching modes. One should note, the signal from B-N skeletal stretching vibrations is represented by a doublet due to natural occurence of two isotopes of boron. Typically as interatomic distances get reduced upon increasing pressure frequencies of measured Raman signals are expected to get higher their values demonstrating a ‘blue’ shift in a measured spectrum. Collected spectra of NaAB, however, revealed an unusual response of B-N and N-H stretching modes to external pressure. While at pressure range from ambient to ~2.1 GPa as well as higher pressure (P > 3 GPa), mode frequencies demonstrated typical behavior – a gradual shift to higher values, a sharp drop in frequency value, ca. 12 and 50 cm^−1^ for B-N and N-H modes respectively, was detected at pressure ~3 GPa (Fig. 1a–d). A shift to a lower frequency (lower energy), known also as a ‘red’ one, is usually a consequence of bond elongation therefore a corresponding mode in vibrational spectrum is called a ‘soft’ one. ‘Soft’ mode behavior of bonds involving hydrogen atoms often points to the enhanced character of dispersive interactions which may lead to formation of either conventional hydrogen or dihydrogen bonds. The observed alternate ‘blue – red – blue’ shift behaviour of N-H modes in the compressed NaAB presents an individual case among metal amidoboranes and pristine ammonia borane. Lithium amidoborane (LiNH_2_BH_3_, LiAB), isostructural with sodium amidoborane, was shown to exhibit a continuous positive pressure dependence (‘blue’ shift) of both symmetric and asymmetric N-H stretching modes up to ~19 GPa. This finding suggests the absence of considerable enhancement of dispersive interactions in the material^15^. In the case of potassium amidoborane (KNH_2_BH_3_, KAB) the crystal structure is similar to that of LiAB and NaAB, however, in addition to molecular fragments [NH_2_BH_3_]^−^ angled at ~45° to *c* axis there is a layer with molecular anions aligned perpendicular to *c* axis. Our recent high-pressure study revealed a spectacular ‘soft’ mode behavior for the one of N-H stretching vibrations indicating formation and continuous enhancement of conventional hydrogen bonding between molecular fragments of different layers^14^. At the same time in pristine ammonia borane the observed ‘soft’ behaviour of all N-H stretching vibrations under pressure is accounted for by the enhancement of dihydrogen bonding, (N-H^δ+^---^δ−^H-B)^16^,^17^. While substantial elongation of N-H bonds resulting from enhancement of dispersive interactions has been observed in KAB and AB, we found no report on possible reason for elongation of skeletal B-N bonds under pressure. We thus undertook an extensive study of high-pressure behaviour of NaAB within *ab initio* theoretical approach with particular attention to bonding properties. Such an approach has been recently shown to yield results consistent with experimental data and proved useful for unveiling complex interplay of bonding in KAB^14^.

**Figure 2:**
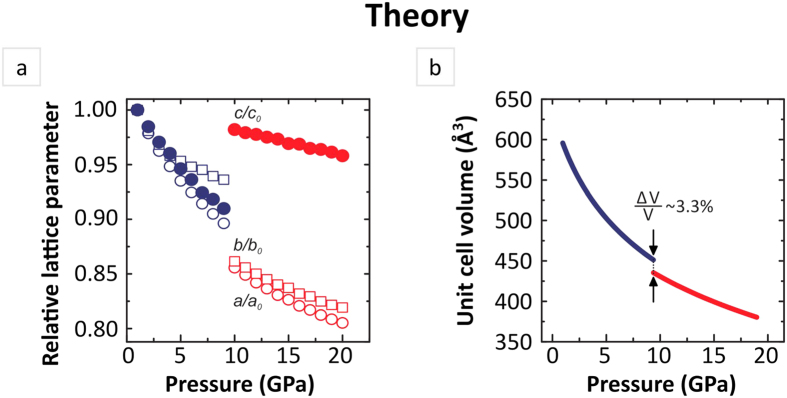
Compressibility of Na(NH_2_BH_3_). (**a**) Effect of pressure on lattice parameters and (**b**) unit cell volume of Na(NH_2_BH_3_). Open symbols represent data of *a* and *b* relative lattice parameters, circle and square, respectively. Pressure evolution of *c* lattice parameter is represented by solid symbols.

For *ab initio* calculations, we used crystal structure parameters obtained by the Rietveld refinement of experimental powder XRD data collected at ambient pressure (See section “Sample characterization” of Supplementary Information). The optimized equilibrium lattice constants and internal atomic positions for initial phase are listed in Table S1 of Supplementary Information. Theoretical lattice parameters for α-NaAB (ambient pressure phase) are in good agreement with the experimental values obtained in our measurements and data reported in the literature^13^. Next the geometry optimization of α-NaAB structure was performed with 1 GPa step in the pressure range up to 20 GPa. It was found the optimized structures at each particular pressure were described within *Pbca* space group. Analysis of the variation of the individual lattice parameters as well as cell volume with pressure, shown in Fig. 2, reveals one of the major findings of this work. Irregular changes of lattice parameters and unit cell volume indicate isosymmetric phase transition at 10 GPa to a new phase, which hereinafter will be called **α**^**/**^-NaAB. Moreover an abnormal expansion of unit cell is found along the *c* axis indicating negative linear compressibility (NLC). The magnitude of NLC can be compared by means of isothermal compressibilities K_NLC_ = (∂lnl/∂p)_T_, defined in terms of the relative rate of change of length ‘l’ with respect to pressure. Further on our calculations demonstrate this material exhibits strong NLC with the magnitude K_NLC_ = −7.9(5) TPa^−1^ which is almost twice as much as values reported for the majority of materials with NLC^1^,^2^,^3^. Abnormal compressibility of NaAB is quite unexpected as its crystal structure reveal no characteristic feature, e.g., tilting/rotating polyhedra networks, helices, specific framework topologies – typically shared by known materials with NLC. We therefore proceeded with detailed analysis of the theoretically optimized structures in order to understand the mechanism of the phase transition calculated at 10 GPa and find out whether the predicted phase transition was of relevance to the changes experimentally observed in Raman spectra.

**Figure 3:**
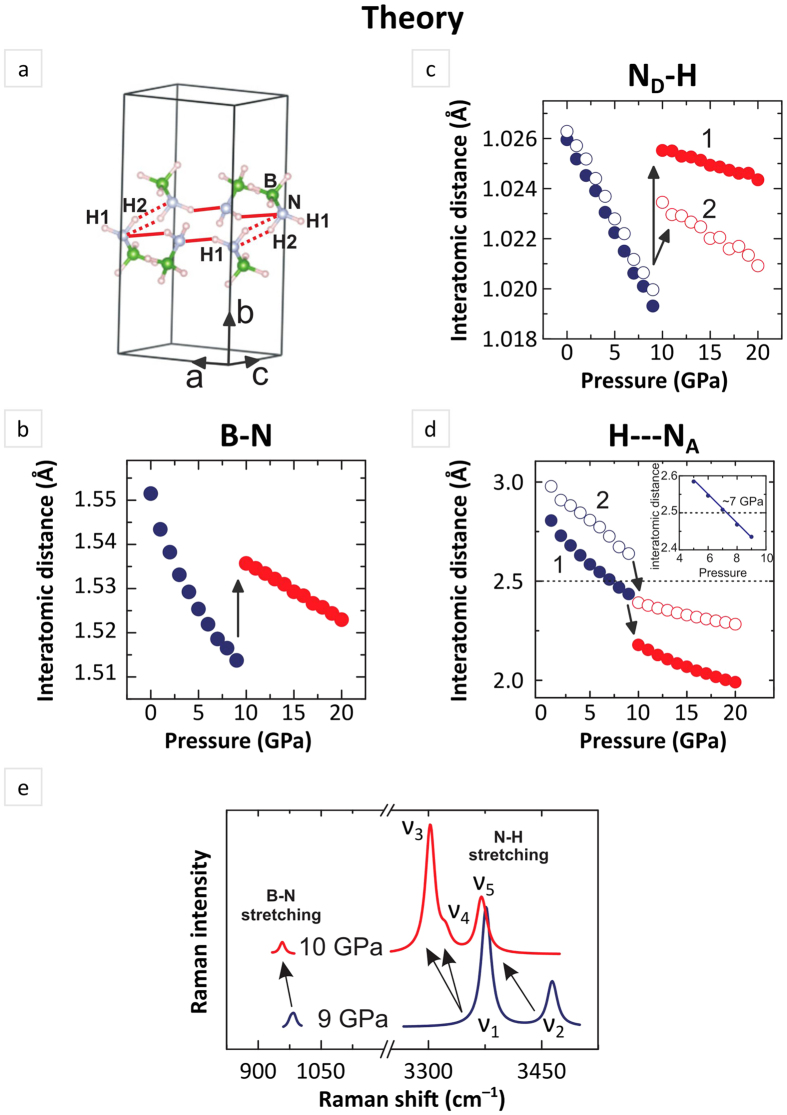
Analysis of possible hydrogen bonds formation based on the calculated pressure effect on interatomic close contacts and illustration of corresponding changes in Raman spectra. (**a**) Fragment view of molecular species, [NH_2_BH_3_]^−^, arrangement illustrating the possible formation of hydrogen bonds of two types: strong and weak one (solid and dot red line, respectively). (**b**) Effect of pressure on B-N, (**c**) N_D_-H intra- and (**d**) N_A_---H intermolecular distances. N_D_ and N_A_ stand for “donor” and “acceptor” atoms of nitrogen, respectively. (**e**) Raman spectra of Na(NH_2_BH_3_) calculated at 9 and 10 GPa in the frequency range of B-N and N-H stretching vibrations. Resolution of calculated spectra is equal to 5 cm^−1^.

In Fig. 3 we plotted pressure effect on intramolecular B-N (d_B-N_), N_D_-H (d_ND-H_), and intermolecular N_A_---H (d_NA---H_) bond lengths. Of note, there are two types of N_D_-H bonds and N_A_---H contacts in the crystal strucure of NaAB. For clarity, plotted data for different N-H contacts engaging H1 and H2 hydrogen atoms are described as **1** and **2** respectively. Up to 9 GPa all the analyzed bonds compress monotonically whereas they exhibit a stepwise counterwise changes at 10 GPa: intramolecular N_D_-H and B-N bond lengths experience substantial elongation whereas intermolecular N_A_---H distance become clearly reduced. Moreover, at pressure above ≥10 GPa, N_D_-H interatomic distances revealed significant anisotropy, in clear contrast to the trend observed at lower pressures. At the same time, the length of N_A_---H contacts became clearly shorter 2.5 Å suggesting possible commencement of stronger dispersive interactions. Pressure-induced changes of bond lengths should have direct implications for vibrational properties of material. We therefore perform comparative analysis for Raman spectra calculated for low- and high-pressure phases of NaAB. Calculated frequencies of Raman modes revealed a clear ‘red’ shift (frequency decrease) in the spectrum at pressure 10 GPa in comparison to those calculated at 9 GPa for both B-N and N-H stretching vibrations. Furthermore spectrum at 10 GPa features higher anisotropy of N-H bonds, where at least three signals from N-H stretching modes can be resolved instead of two calculated at 9 GPa (details of mode assignments for calculated spectra are presented in Figure S3 of Supplementary Information).

**Figure 4:**
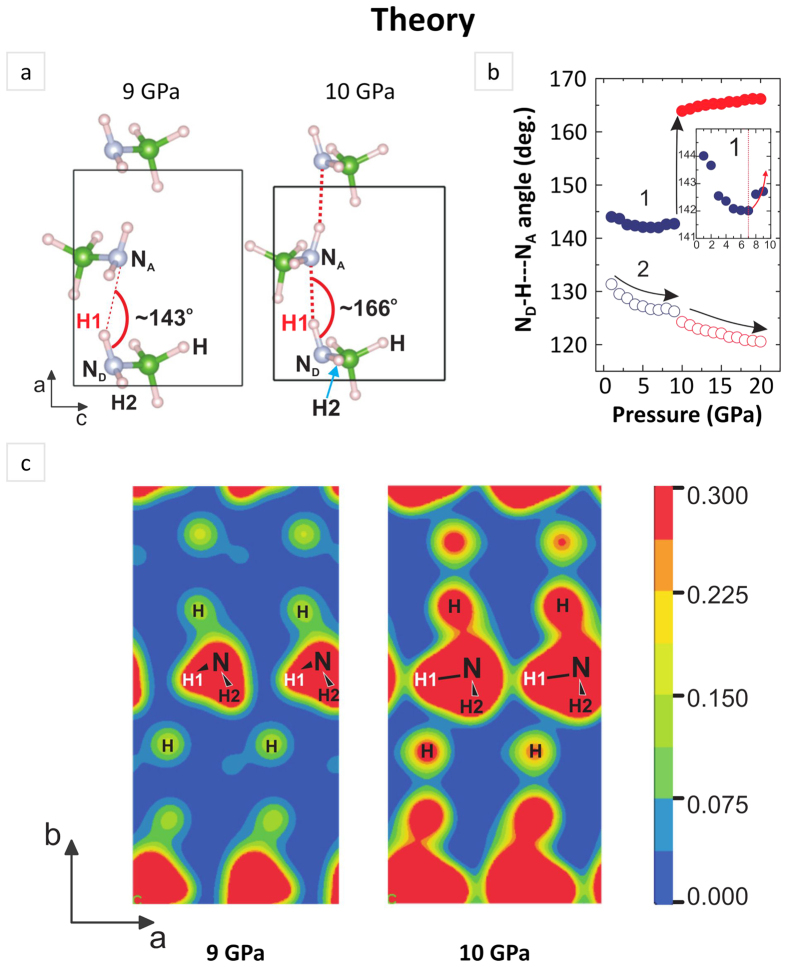
Detailed analysis of hydrogen bond formation and its effect on charge density distribution. (**a**) Arrangement of molecular anions [NH_2_BH_3_]^−^ in the cell viewed along *b* axis at 9 GPa and 10 GPa. Sodium cations and the rest of molecular species are omitted for clarity. N_D_ and N_A_ – define donor and acceptor nitrogen atom respectively; H stands for hydrogen atoms bound to boron atoms (green sphere); dotted line illustrates hydrogen bonding (**b**) Change of ∠N_D_-H---N_A_ angle with pressure. (**c**) Charge density distribution of NaAB within the scale of 0 to 0.300 e/Å^3^ calculated at 9 and 10 GPa.

To further analyze the nature of chemical bonding we plot the variation of ∠N_D_-H---N_A_ angle with pressure (Fig. 4). Depending on hydrogen atom under consideration data for molecular fragments engaging H1 and H2 are described as **1** and **2** respectively. One can note a remarkably different effect of pressure on angle trends **1** and **2** across the phase transition. The molecular fragments engaging hydrogen atoms H1, in clear contrast to those with H2, experience considerable flattening, increase of ∠N_D_-H---N_A_ values, corraborating possible formation of hydrogen bond. To probe the existence of hydrogen bonding in **α**^**/**^-NaAB, we calculated the charge density distribution (CDD) as well. Distribution of charge mapped within the plane parallel to *a* and *b* axes sliced across NH_2_ groups for α- (at 9 GPa) and **α**^**/**^-NaAB (at 10 GPa) are displayed in Fig. 4. According to the calculated CDD dominating interaction between N and H atoms is a strong polarized covalent bond in both phases. At the same time, an increased charge density between the nearest N and H1 atoms belonging to neighboring NH_2_ group indicates the possibility of the existence of hydrogen bonding in **α**^**/**^-NaAB, in contrast to low values of the electron density in interstices of the NH_2_ groups found in α-NaAB.

Data presented in Figs 3 and 4 imply that the new phase, **α**^**/**^-NaAB, has rather strong N_D_-H---N_A_ hydrogen bonds, engaging H1 type of hydrogen atoms, which account for the major changes in calculated Raman spectra. The formation of hydrogen bonds engaging H2 hydrogen atoms cannot be ruled out since the H---N_A_ intermolecular distance (data set **2** in the Fig. 3(d)) reaches values < 2.5 Å at 10 GPa as well. However, gradual drop of ∠N_D_-H---N_A_ values (data set **2** in the Fig. 4(b)) indicates rather weakening of hydrogen bonds of this type with further pressure increase.

**Figure 5:**
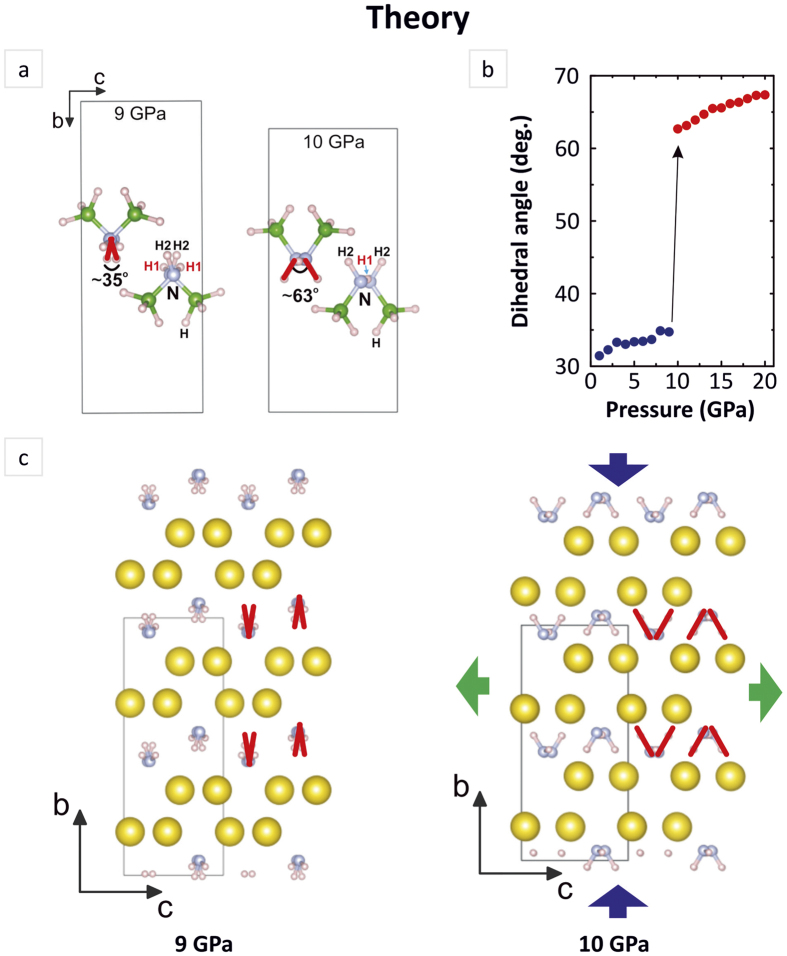
The schematic illustration of pressure effect on overall crystal geometry and the particular arrangement of ‘NH_2_’ molecular fragments. (**a**) Diagrammatic representation of ‘N-H’ bonds (engaging hydrogen atoms of H2 type) as red rods forming dihedral angle at pressure 9 and 10 GPa (viewed along *a* axis) (**b**) Variation of the dihedral angle with pressure (**c**) Crystal packing of the low-pressure form viewed along the *a* axis at 9 GPa and at 10 GPa, where the blue and green arrows show directions of compression and expansion respectively. BH_3_ molecular fragments are omitted for clarity.

Following the spectroscopic changes observed both experimentally and computationally, the NLC mechanism of NaAB can be rationalized as follows. Hydrostatic compression leads to distance reduction (d_NA---H_) between neighbour NH_2_ groups which significantly enhance initially weak intermolecular interactions. At the pressure about 7 GPa the d_NA---H_ distance approaches the critical value of 2.5 Å driving the formation of chains of weak hydrogen bonds. Compression to about 9 GPa leads to gradual evolution of weak hydrogen bonds to a moderate ones which is featured by both gradual increase of ∠N_D_-H---N_A_ angle values and gradual shortening of the d_NA---H_ distance. Further enhancement of intermolecular hydrogen bonding is featured by an abrupt and substantial reduction of the d_NA---H_ distance along with flattening of N_D_-H---N_A_ fragments. Enhanced chemical attraction between neighbour molecular moieties results in elongation and thus weakening strength of B-N skeletal bonds which should be evidenced by the ‘red’ shift in Raman spectrum. Weaker B-N bonds facilitate rotation of NH_2_ groups, corraborated by the steep increase of dihedral angle, in the plane perpendicular to N_D_-H---N_A_ bond line which gives rise to negative linear compressibility of crystal along *c* axis (Fig. 5).

Similar character of observed changes in experimental Raman spectra, namely, the “redshift” of B-N and N-H stretching modes as well as the higher anisotropy of N-H stretching vibrations, strongly suggests the experimentally found phase transition at about 3 GPa is of the same origin with the computed one at 10 GPa. The discrepancy in the value of the phase transition pressure might stem from the fact that total-energy calculations were performed at zero temperature. The measured magnitude of the “redshift”: ~14 and 45 cm^−1^, is lower than the calculated one: 25 and 74 cm^−1^ for B-N and N-H stretching modes respectively. The magnitude of the N-H “redshift” is a measure of hydrogen bond strength. Assuming there is a linear dependence of the expansion rate along *c* axis on the magnitude of the N-H “redshift” the material exhibit one of the strongest NLC reported in the literature.

As a matter of speculation, the observed high-pressure behaviour of NaAB may have implications suitable for practical uses. Due to elongation of interatomic distance the strength of N-H bonds in ***α***^**/**^-NaAB is expected to be lower than in *α*-NaAB. Therefore the weakening of N-H bonds may facilitate the hydrogen release from ***α***^**/**^-NaAB. Considering the low pressure of *α*- to *α*^**/**^-NaAB phase transition one may attempt recovering of ***α***^**/**^-NaAB to ambient pressure through additional modification of chemical composition, *i.e*., either substitution or doping, like it has been demonstrated for materials based on cyanide framework^18^,^19^. Considering the narrow pressure range of the phase transition, perhaps, the most immediate application of NaAB, beside hydrogen storage, would be a pressure sensitive switchable component of a device using NLC. Similarly one can speculate certain modification of chemical composition may have beneficial effect on the magnitude of NLC through tuning subtle interactions in NaAB.

In conclusion, a comprehensive high-pressure study of structural properties of sodium amidoborane (NaNH_2_BH_3_, NaAB) was performed using *in situ* Raman spectroscopy measurements and density functional theory calculations. Particular attention was drawn to understanding the role of dispersive interactions in structural stability of NaAB. In our calculations the isosymmetric phase transition to ***α***^**/**^-NaAB phase was found at 10 GPa. Experimental proofs of the transition were found at pressure about 3 GPa. The phase transition is accompanied by a strong negative linear compressibility (NLC) along crystal *c* axis. The first of its kind the mechanism of the observed NLC is controlled by the pressure-induced enhancement of dispersive interactions which leads to formation of hydrogen bonds. This work uncovers an example of intricate interplay between chemical interactions and mechanical response which opens up an exciting new direction for tailoring materials with rare functionality.

## Methods

### Sample preparation

We used the highest purity available substrates: NH_3_BH_3_ (98%, JSC Aviabor), NaH (95%, Sigma Aldrich) and THF (99.9%, Sigma Aldrich). We synthesized sodium amidoborane (NaAB) in a direct reaction of ammonia borane with sodium hydride in solid state *via* a dry mechanochemical way described in the literature^13^,^20^,^21^, using tungsten carbide disk milling vessel together with a high energy mill from Testchem. All operations were carried out under argon atmosphere with no contact with atmospheric air, according to the reaction equations:


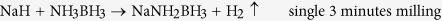


In an alternative way we synthesized NaAB in a direct reaction of sodium hydride with THF solution of ammonia borane. We used dry THF as a solvent under argon atmosphere with no contact with atmospheric air, according to the reaction equations:


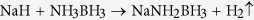


After the reaction the solvent was desorbed at room temperature under argon atmosphere.

### Variable-pressure *in situ* Raman scattering measurements

Diamond anvil cell (DAC) with type-I diamonds of 500 μm culet size was used for the high-pressure experiments. A stainless steel gasket was preindented to ~ 35 μm thicknesses and then a hole of ~155 μm in diameter was drilled in the center as a sample chamber. Polycrystaline sample, powder of NaAB, along with some ruby crystals (in form of sphere) for *in situ* pressure measurement, was loaded in the sample chamber inside the glovebox. Pressure was calibrated from the shift of Ruby fluorescence. Several series of measurements upon pressure loading and unloading course were performed. Except one run, no pressure medium was used in the sample chamber, because sample material is fairly soft. For comparison, one run of measurements was performed with mineral oil as a pressure medium. The results showed no noticeable difference in the high-pressure behavior of NaAB.

Raman spectra of NaAB loaded in DAC were collected in backscattered geometry using custom disigned setup for micro-Raman measurements based on monochromator Jobin Yvon THR1000 (focal length 1000 mm) equipped with a single grating (with 1200 grooves mm^−1^) giving a resolution of ~1 cm^−1^, notch filters (Keiser Optical Systems) and thermoelectrically cooled (−75 °C) (Peltier effect) CCD (Horiba Synapse) detection. He-Ne laser (Melles-Griot) line 632.8 nm was used for sample excitation.

### Details of *ab initio* calculations

The underlying *ab initio* structural relaxations and electronic calculations have been carried out using density functional theory within the Perdew−Burke−Ernzerhof (PBE) exchange correlation as implemented in the CASTEP code^22^. Lattice parameters and atomic positions were optimized by seeking a total minimum energy based on density-functional theory and the plane-wave pseudopotential method. In order to perform the geometry optimization, the following thresholds were applied for a convergence window of two successive self-consistent steps: total energy change smaller than 5 × 10^−6^ eV/atom, maximum force per atom below 0.01 eV/Å, pressure smaller than 0.02 GPa, and maximum atomic displacement not exceeding 5 × 10^−4^ Å. The BFGS minimizer was employed to carry out the unit cell optimization. The quality of this basis set was kept fixed as the unit cell volume varied during geometry optimization. The cutoff energy of 770.0 eV for the expansion of the wave function into plane waves and fine Monkhorst− Pack k meshes have been chosen to ensure that all the energy calculations are well converged to required values.

## Additional Information

**How to cite this article**: Magos-Palasyuk, E. *et al*. Chemically driven negative linear compressibility in sodium amidoborane, Na(NH_2_BH_3_). *Sci. Rep*. **6**, 28745; doi: 10.1038/srep28745 (2016).

## Supplementary Material

Supplementary Information
