# Supplementary material for: Chemically driven negative linear compressibility in sodium amidoborane, Na(NH2BH3)
Source: Sci Rep. 2016 Jun 30;6:28745. doi: 10.1038/srep28745 (PMC4928083; doi:10.1038/srep28745)
Supplement: Supplementary Information [file srep28745-s1.doc]

SUPPLEMENTARY INFORMATION

**Chemically driven negative linear compressibility in sodium amidoborane, Na(NH_2_BH_3_)**

Ewelina Magos-Palasyuk*^a^*, Karol J. Fijalkowski*^b^* and Taras Palasyuk**^a^*

^a^ Institute of Physical Chemistry PAS, Kasprzaka Str. 44/52, Warsaw, Poland.

e-mail: tpalasyuk@ichf.edu.pl

^b^ Centre of New Technologies, University of Warsaw, S. Banacha Str. 2c, 02-097 Warsaw, Poland.

**TABLE OF CONTENTS**

**1. Sample characterization**

**2.** **Table of crystal structure parameters**

**3. Normal mode analysis of N – H stretching vibrations**

**1. Sample characterization**

Powder X-ray diffraction (PXRD) patterns of sodium amidoborane (NaAB) sealed in 0.5 quartz capillaries were measured using Bruker D8 Discover diffractometer with 2D Vantec detector (parallel beam; the CuK_α1_ and CuK_α2_ radiation intensity ratio of *ca.* 2:1, λ ~ 1.5406 Å). The Rietveld plot of NaAB is presented in Figure S1.


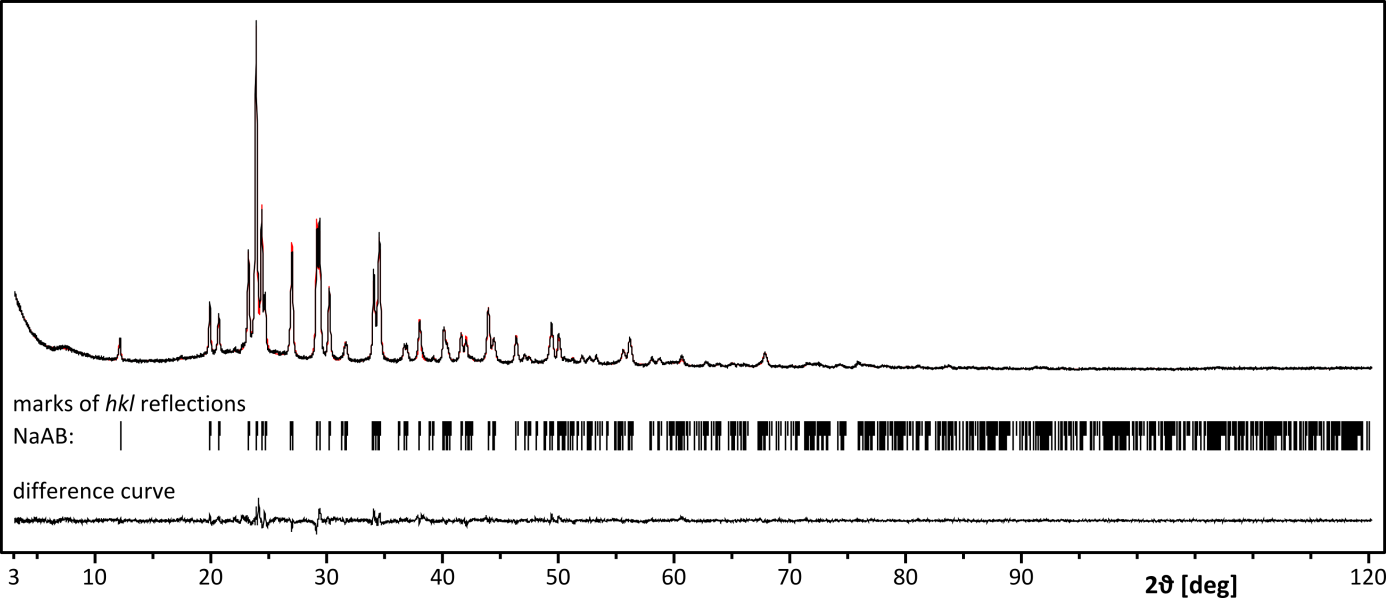


**Figure S1.** Powder X-ray diffraction pattern of NaAB sample taken at ambient pressure at room temperature. Refined lattice parameters a =7.46930 (Å); b = 14.63980 (Å); c = 5.65720 (Å).

**2. Table of crystal structure parameters**

**Table S1.** Calculated results of variable-pressure relaxation of NaAB crystal structure.

| **Pressure** | **Structure type**  **(*space group*)** | **Lattice parameters (Å)** | **Atomic coordinates (fractional)** | | | |
| --- | --- | --- | --- | --- | --- | --- |
|  |  |  | **Element** | ***x*** | ***y*** | ***z*** |
| ambient | α – NaNH_2_BH_3_  (*Pbca*) | *a* = 7.5740  *b* = 14.8346  *c* = 5.7686 | Na: | -0.04209 | 0.16823 | 0.43131 |
|  |  |  | B: | 0.08886 | 0.14652 | 0.96530 |
|  |  |  | N: | 0.06898 | 0.08299 | 0.75358 |
|  |  |  | H1 (B)**^a^**: | 0.15927 | 0.11144 | 1.14026 |
|  |  |  | H2 (B): | 0.17935 | 0.21285 | 0.91187 |
|  |  |  | H3 (B): | -0.05851 | 0.17424 | 1.02621 |
|  |  |  | H1 (N): | -0.00203 | 0.02623 | 0.78841 |
|  |  |  | H2 (N): | 0.18968 | 0.05932 | 0.70051 |
| 9 GPa | α – NaNH_2_BH_3_  (*Pbca*) | *a* = 6.60390  *b* = 13.34040  *c* = 5.16120 | Na: | -0.02331 | 0.16915 | 0.43717 |
|  |  |  | B: | 0.09749 | 0.13780 | 0.94490 |
|  |  |  | N: | 0.09305 | 0.05748 | 0.73781 |
|  |  |  | H1 (B): | 0.17919 | 0.11383 | 1.15132 |
|  |  |  | H2 (B): | 0.19122 | 0.21079 | 0.86085 |
|  |  |  | H3 (B): | -0.07582 | 0.16484 | 0.99743 |
|  |  |  | H1 (N): | 0.01349 | -0.00506 | 0.78841 |
|  |  |  | H2 (N): | 0.23293 | 0.03346 | 0.68199 |
| 10 GPa | α**/** – NaNH_2_BH_3_  (*Pbca*) | *a* = 6.3049  *b* = 12.2744  *c* = 5.5713 | Na: | 0.01566 | 0.14271 | 0.45942 |
|  |  |  | B: | 0.11011 | 0.14261 | 0.94064 |
|  |  |  | N: | 0.13321 | 0.04001 | 0.78508 |
|  |  |  | H1 (B): | 0.19390 | 0.13641 | 1.13781 |
|  |  |  | H2 (B): | 0.19695 | 0.21712 | 0.83568 |
|  |  |  | H3 (B): | -0.07538 | 0.16757 | 0.97672 |
|  |  |  | H1 (N): | 0.10255 | -0.02977 | 0.87946 |
|  |  |  | H2 (N): | 0.28846 | 0.03161 | 0.73339 |

**^a^** a hydrogen atom makes a covalent bond with an element in parenthesis

**3. Normal mode analysis of N – H stretching vibration**

**Figure S3.** Assignment of the N – H stretching modes of NaAB Raman spectra calculated at 9 and 10 GPa (color coded in blue and red respectively).
